# Role of ATP-sensitive potassium channels on hypoxic pulmonary vasoconstriction in endotoxemia

**DOI:** 10.1186/s12931-018-0735-x

**Published:** 2018-02-13

**Authors:** Maurizio Turzo, Julian Vaith, Felix Lasitschka, Markus A. Weigand, Cornelius J. Busch

**Affiliations:** 1 Department of Anesthesiology, Heidelberg University Hospital, Im Neuenheimer Feld 110, 69120 Heidelberg, Germany; 2 Institute of Pathology, Heidelberg University Hospital, Heidelberg, Germany

**Keywords:** Hypoxic pulmonary constriction, Endotoxemia, Lung, Mouse, Kir6.1

## Abstract

**Background:**

ATP-regulated potassium channels (KATP) regulate pulmonary vascular tone and are involved in hypoxic pulmonary vasoconstriction (HPV). In patients with inflammation like sepsis or ARDS, HPV is impaired, resulting in a ventilation-perfusion mismatch and hypoxia. Since increase of vascular KATP channel Kir6.1 has been reported in animal models of endotoxemia, we studied the expression and physiological effects of Kir6.1 in murine endotoxemic lungs. We hypothesized that inhibition of overexpressed Kir6.1 increases HPV in endotoxemia.

**Methods:**

Mice (C57BL/6; *n* = 55) with (*n* = 27) and without (*n* = 28) endotoxemia (35 mg/kg LPS i.p. for 18 h) were analyzed for Kir6.1 gene as well as protein expression and HPV was examined in isolated perfused mouse lungs with and without selective inhibition of Kir6.1 with PNU-37883A. Pulmonary artery pressure (PAP) and pressure-flow curves during normoxic (F_i_O_2_ 0.21) and hypoxic (F_i_O_2_ 0.01) ventilation were obtained. HPV was quantified as the increase in perfusion pressure in response to hypoxic ventilation in mmHg of baseline perfusion pressure (ΔPAP) in the presence and absence of PNU-37883A.

**Results:**

Endotoxemia increases pulmonary Kir6.1 gene (+ 2.8 ± 0.3-fold) and protein expression (+ 2.1 ± 0.3-fold). Hypoxia increases HPV in lungs of control animals, while endotoxemia decreases HPV (∆PAP control: 9.2 ± 0.9 mmHg vs. LPS: 3.0 ± 0.7 mmHg, *p* < 0.05, means ± SEM). Inhibition of Kir6.1 with 1 μM PNU-37883A increases HPV in endotoxemia, while not increasing HPV in controls (∆PAP PNU control: 9.3 ± 0.7 mmHg vs. PNU LPS: 8.3 ± 0.9 mmHg, *p* < 0.05, means ± SEM).

**Conclusion:**

Endotoxemia increases pulmonary Kir6.1 gene and protein expression. Inhibition of Kir6.1 augments HPV in murine endotoxemic lungs.

## Background

Hypoxic pulmonary vasoconstriction (HPV) is a physiological reflex, reducing intrapulmonary shunt. It is impaired in patients with sepsis or acute respiratory distress syndrome (ARDS), resulting in a ventilation-perfusion mismatch and systemic hypoxia. Reduced HPV is not only restricted to critical ill humans but can also be observed in several animal models of endotoxemia [1–3]. Inflammatory mediators including prostaglandins, thromboxanes, platelet-activating factor, leukotrienes, or nitric oxide (NO) modulate HPV during lung inflammation [4]. Voltage gated potassium channels have been shown to be one of the key regulators of HPV [5], but in candidates like Kv1.5, Kv2.1 or Kv3.1, no alteration in expression levels in endotoxemia was found [3], despite unspecific inhibition with 4-AP augments HPV. In contrast, ATP sensitive K-channels (KATP) were demonstrated to be increased in endotoxemia [6]. They are also involved in regulation of pulmonary vascular tone and HPV in various organisms, including mice, rats and pigs [7, 8]. Inhibition of KATP channels has been shown to increase pulmonary artery resistance [8].

The K-channel Kir6.1 (potassium inwardly-rectifying channel, subfamily J, member 8) is encoded by the gene kcnj8. The channel is built by an octameric protein complex consisting four pore-forming Kir6.1 units surrounded by four sulfonylurea receptor subunits (SURs). These SURs present binding sites for inhibitors like glibenclamid or PNU-99963, while the pore forming units can be blocked by barium chloride or the vascular selective KATP channel inhibitor, PNU-37883A [6, 9].

Expression of the KATP-channel Kir6.1 has been shown in smooth muscle cells of pulmonary arteries, the mouse aorta, rat mesenteric arteries, cerebral and coronary arteries as well as urethral myocytes [10, 11]. Furthermore, KATP-channels have been described in rat and bovine pulmonary vascular endothelial cells as well as human pulmonary artery smooth muscle cells [12, 13]. Activation of KATP-channels increases efflux of K^+^, resulting in increased closure of Ca^2+^ channels, decreased Ca^2+^-influx and reduced intracellular Ca^2+^, which in turn leads to smooth muscle relaxation. In endotoxemia, Kir6.1 has been shown to be up-regulated in mouse aortic smooth muscle cells and attributed to systemic hypotension [11].

We report that Kir6.1 expression was induced in endotoxemic mouse lungs on mRNA as well as protein level. Immunohistochemistry showed a Kir6.1 immunoreactive protein in pulmonary mouse veins and arteries. Selective inhibition of Kir6.1 resulted in augmented HPV in isolated perfused endotoxemic mouse lungs.

## Methods

A total of 55 adult male C57BL/6 mice (Charles River GmbH, Sulzfeld, Germany) with 8–10 weeks of age and 23.0 ± 0.2 g body weight (bw) were studied.

### Experimental groups

Mice received an intraperitoneal (i.p.) injection of endotoxin (LPS; E.coli 0111:B4, 35 mg/kg bw; Sigma Chemical Co., St. Louis, MO) in LPS groups (*n* = 7 per group) or normal saline (controls, *n* = 8 per group) 18 h before isolated lung perfusion experiments. Animals showed lethargy, piloerection, and diarrhea 18 h after LPS injection, mortality rate was approximately 10%. For lung perfusion, PNU-37883A (Sigma Chemical) was dissolved in ethanol and added to give a final concentration in the perfusate of 0 or 1 μM PNU-37883A in the respective groups. The ethanol concentration used in the perfusates was 0.5 mg/dl.

### Isolated, perfused, and ventilated mouse lung model

Mice were sacrificed by an i.p. injection of pentobarbital sodium (200 mg/kg body weight) (Merial, Hallbergmoos, Germany) and lungs were explanted and buffer perfused as previously described [3]. Perfusate flow was adjusted using an in-line flow probe and flowmeter (Transonic Systems, Ithaca, NY). The perfusate used was Hanks’ balanced salt solution (Life Technologies, Paisley, Scotland), with bovine serum albumin (5%; Serva, Heidelberg, Germany) and dextran (5%; Sigma-Aldrich Chemie, Deisenhofen, Germany) added to prevent pulmonary edema [3]. Indomethacin (30 mM; Sigma-Aldrich) and 1 mM of the nonselective nitric oxide synthase inhibitor L-NAME were added to the perfusate to inhibit endogenous prostaglandin synthesis and nitric oxide synthesis respectively. PNU-37883A was added to the perfusate to give a final concentration of 0 or 1 μM and stirred before lung perfusion. Sodium bicarbonate was added to adjust the perfusate pH (7.34–7.43). Lungs were included in this study if they had a homogenous white appearance without signs of hemostasis or atelectasis and showed a stable perfusion pressure less than 10 mmHg during the second 5 min of an initial 10 min baseline perfusion period. Using these two criteria, approximately 10% of lung preparations from each group were discarded before study. Pulmonary artery pressure (PAP) and left atrial pressure were measured via saline-filled membrane pressure transducers connected to a side port of the inflow and outflow cannula. Pressure transducers were connected to a biomedical amplifier, and data were recorded at 150 Hz on a personal computer using an analog-to-digital interface with a data acquisition system (DI-220; Dataq Instruments, Akron, OH). The system was calibrated before each experiment. HPV responsiveness (ΔPAP) was quantified as the difference between basal pulmonary arterial pressure (PAP) and PAP at the end of six minute ventilation at F_i_O_2_ of 0.01.

Pulmonary vascular pressure-flow relationships were obtained during normoxia and hypoxia. After a 10 min equilibration period with normoxic ventilation (F_i_O_2_ = 0.21) and a perfusate flow of 50 ml*kg-1*min-1, flow was set to 25, 50, 75, and 100 ml*kg-1*min-1 in a randomized fashion for 30 s each and the corresponding PAP recorded. Then, ventilation was switched to hypoxic gas (F_i_O_2_ = 0.01, perfusate flow 50 ml*kg-1*min-1) for measurement of ΔPAP and a second pressure-flow relationship was recorded in the same manner at the end of a six minute ventilation at F_i_O_2_ of 0.01.

Pulmonary vascular pressure-flow relationships were analyzed using a linear distensible vessel model as described before [15]. Briefly, this model describes the vascular pressure-flow characteristic using two parameters: R_LIN_ is interpreted as mean parallel resistance extra-alveolar, non-collapsible pulmonary vessels and P_ZF_ as mean critical closing pressure, representing a mean pressure value below a pressure that would not result in a flow in the pulmonary vessels.

### Lung wet /dry weight ratio

At the end of the experiments, both lungs of the studied animals, excluding their hilar structures, were excised and immediately weighed. Thereafter, lungs were dried in an oven at 100 °C overnight and then re-weighted. Lung wet/dry weight ratios were calculated by dividing the wet weight by the dry weight as described previously [16].

### Semi-quantitative RT-PCR

Additional animals (*n* = 18) were used for isolation of lung tissue for RNA and protein analysis. After 18 h of LPS exposure (LPS; E.coli 0111:B4, 35 mg/kg bw i.p.; Sigma Chemical Co., St. Louis, MO), mice were sacrificed with a lethal i.p. injection of pentobarbital sodium (200 mg/kg bw, three independent experiments with *n* = 3 each). Saline injected mice served as controls (also three independent experiments with *n* = 3 each). Lungs were exposed via median sternotomy and heparin (10 U) was injected into the right ventricle. Lungs were perfused with iced physiological saline for one minute at 50 ml/kg-1/min-1 flow, dissected (excluding hilar structures), quick-frozen, and stored at − 80 °C.

RNA was isolated from mouse lungs using the RNeasy Mini Kit (Qiagen) and cDNA was generated with iScript™ cDNA Synthesis Kit (Bio-Rad Laboratories, Herkules, USA). Semi-quantitative PCR was carried out on a MyiQ Single-Color Real-Time PCR detection system (Bio-Rad Laboratories, Herkules, USA), using specific primers for Kir6.1 (FP: GGCACACAAGAACATCCGAGAG, RP: TGCAGAGGAAGGACATGGTGA) and 18S (FP: TCAAGAACGAAAGTCGGAGG, RP: GGACATCTAAGGGCATCAC). Postamplification dissociation curves were performed to verify the presence of a single amplification product in the absence of DNA contamination. Changes in gene expression were determined using the ∆∆Ct method with normalization of 18S ribosomal RNA.

### Immunoblotting

Western blots were performed to assess protein levels of Kir6.1 and GAPDH. In brief, mouse lungs with and without 18 h endotoxemia (three independent experiments with *n* = 3 each) were homogenized at 4 °C in PBS with 5 mM EGTA and protease inhibitor mix (Roche Diagnostics GmbH, Germany) and centrifuged at 10,000 g and 4 °C for 10 min. Supernatant protein was subjected to electrophoresis, transferred to a PVDF membrane, blocked for 1 h at room temperature with i-Block 0,5% and probed with anti-Kir6.1 (1:500, Alomone, Jerusalem, Israel) and anti-GAPDH (1:10,000, Millipore, Darmstadt, Germany) overnight at 4 °C. For negative control, 1 μg of purified Kir6.1 control peptide antigen (Alomone) was preincubated with 1 μg of antibody for one hour at room temperature and then incubated with the membrane overnight at 4 °C. The PVDF membranes were then incubated with corresponding secondary antibodies (1:10,000 IRDye 680 and 800, LI-COR Biotechnology, Lincoln, USA). Proteins were visualized with a LI-COR infrared imager (Odyssey, LI-COR Biotechnology), quantitative densitometric analysis was performed by applying Odyssey version 1.2 infrared imaging software and signals were normalized to GAPDH. Coincubation with the Kir6.1 control peptide antigen faded away the Kir6.1 immunoreactive band at a size of 50 kDa.

### Immunoenzyme staining

Lungs of mice with (*n* = 4) and without (*n* = 4) 18 h of endotoxemia were fixed in paraformaldehyde. Immunoenzyme stainings were performed on 2 μm paraffin-embedded sections using standard avidin-biotin anti-alkaline phosphatase technique (Vector Laboratories, Burlingame, CA) according to the manufacturer’s instructions. Tris-buffered saline supplemented with 0.2% bovine serum albumin (Biotrend, Cologne, Germany) was used as buffer. Primary antibody dilutions of polyclonal rabbit anti-Kir6.1, 1/50 (Alomone) and an isotype- and concentration-matched rabbit control Ig (Dianova, Hamburg, Germany) were prepared in this buffer and incubated for 1 h at room temperature. A biotinylated donkey anti-rabbit IgG Ab, 1/100 (Jackson ImmunoResearch, Newmarket, UK), was used as a secondary reagent (30 min at room temperature). Naphthol AS-biphosphate (Sigma) with New-fuchsin (Merck, Darmstadt, Germany) was used as the substrate for alkaline phosphatase.

### Statistical analysis

Data are reported as mean ± SEM. After approving the assumption of normality and equal variance across groups, differences were assessed using ANOVA followed by an appropriate post hoc comparison test. When significant differences were detected by ANOVA, a post hoc least difference test for planned comparisons was used (SPSS 24, IBM, Armonk, USA). Statistical significance was assumed at a *p* value of less than 0.05.

## Results

### Endotoxemia increases pulmonary Kir6.1 gene expression

**Fig. 1:**
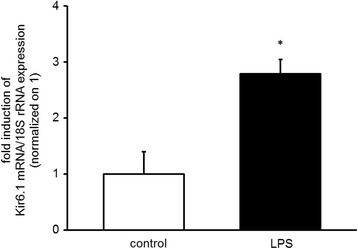
Endotoxemia increases Kir6.1 gene expression in mouse lungs. RNA was extracted from mice with and without endotoxemia (rRNA: ribosomal RNA, representative QPCR, *n* = 3, means ± SEM, *P < 0.05 vs. control, tested with ANOVA, for homogeneity and post hoc Tukey)

Mice exposed to 35 mg/kg LPS i.p. showed an increase of Kir6.1 gene expression in total lung tissue extracts (2.8 ± 0.3-fold, *n* = 9, *p* < 0.05) (Fig. 1) after 18 h when compared to normal saline treated controls. These results suggest that endotoxemia increases RNA expression of the ATP-dependent potassium channel Kir6.1.

### Endotoxemia increases Kir6.1 protein expression in the lung

**Fig. 2:**
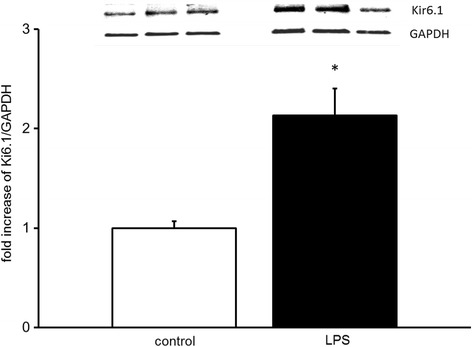
Endotoxemia increases Kir6.1 protein expression in mouse lungs. Densitometric measurement of the Kir6.1 immunoreactive protein levels is shown. (Representative immunoblot, *n* = 3, normalized to GAPDH, control normalized on 1, means ± SEM, *p < 0.05, tested with ANOVA, for homogeneity and post hoc Tukey)

To test whether up-regulation of Kir6.1 gene expression results also in increased protein expression, tissue of mouse lungs was extracted after 18 h of endotoxemia, saline injected animals served as controls. Kir6.1 immunoreactive protein was increased in whole lung extracts (2.1 ± 0.3-fold, *n* = 9, *p* < 0.05, mean ± SEM, Fig. 2). These results confirm that endotoxin challenge provokes an induction of Kir6.1 protein expression.

### Kir6.1 expression is located pulmonary vessels

**Fig. 3:**
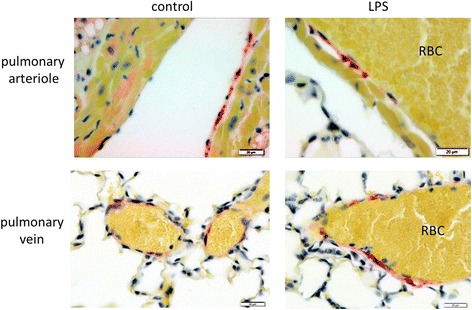
Kir6.1 immunoreactivity in lungs of control mice (left) and endotoxemic mice (right). Immunoenzyme stainings were performed on paraffin-embedded sections using polyclonal rabbit anti-Kir6.1 1:50 (Alomone). Endothelial cells as well as smooth muscle cells of small pulmonary vessels show positive staining in control as well as endotoxemic animals. RBC: red blood cells

To evaluate location of Kir6.1 protein expression, lungs of mice (*n* = 4) with and without 18 h of endotoxemia where fixed in paraformaldehyde, paraffin-embedded and stained with Kir6.1 specific antibodies (Fig. 3). Kir6.1 positive staining was found in the wall of small pulmonary arteries and veins. Region of interest of magenta positive area (equals Kir6.1 positive staining) was 0.41 ± 0.29% in controls vs. LPS 0.60 ± 0.29% (normalized on 1 control: 1.0 ± 0.29 vs. LPS 1.48 ± 0.51; *p* = 0.03, mean ± SEM). These results show that Kir6.1 expression is attributable to pulmonary vessels.

### Pulmonary vascular response to hypoxic ventilation after lipopolysaccharide challenge

**Fig. 4:**
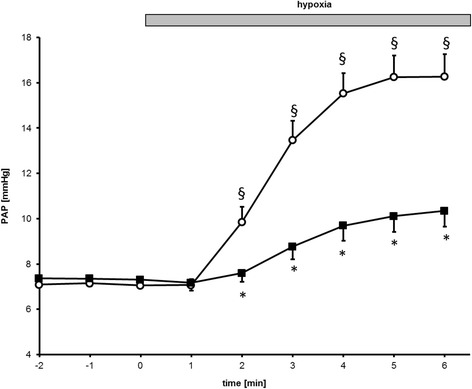
Endotoxemia decreases HPV in isolated perfused mouse lungs. Time course of pulmonary artery pressure at normoxic baseline (− 2 to 0 min) and during hypoxic ventilation (1–6 min; hypoxia) in lungs isolated from LPS-pretreated (■) and untreated control mice (□) (*n* = 8 for controls, *n* = 7 for LPS,*P < 0.05 vs. corresponding control, §*P* < 0.05 vs. control normoxia, mean ± SEM, tested with ANOVA, for homogeneity and post hoc Tukey)

**Fig. 5:**
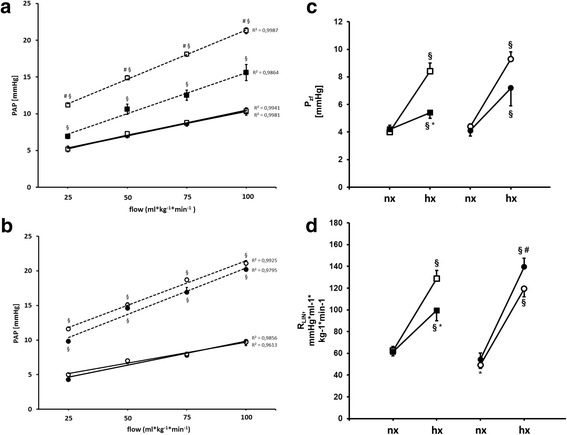
Pressure-flow data obtained in isolated, perfused mouse lungs of untreated and LPS treated mice (**a**) (*n* = 8 for controls (□) and *n* = 7 for LPS (■), trend lines). Lungs were isolated and perfused with a flow of 25, 50, 75, and 100 ml* kg − 1 * min − 1 during ventilation with a normoxic (straight line) or hypoxic gas mixture (dashed line), respectively, and the resulting perfusion pressure (PAP) was recorded. **b**: Pressure-flow relationships were obtained under perfusion with 1 μM PNU 37883A (*n* = 7 for PNU (○) and *n* = 7 for LPS/PNU (●), straight line normoxic gas, dashed line hypoxic gas. **c**: P_zf_ increases upon hypoxic ventilation as well as R_LIN_ (**d**). Perfusion with 1 μM PNU-37883A increased P_zf_ as well as R_LIN_ during hypoxic ventilation (Fig. 5c and d). (nx: normoxia, hx: hypoxia, *P < 0.05 vs. corresponding control, # vs. LPS, § vs corresponding normoxia; mean ± SEM, tested with ANOVA, for homogeneity and post hoc Tukey)

Hypoxic ventilation of lungs of control mice caused an HPV response (∆PAP: + 9.2 ± 0.9 mmHg, Fig. 4). Accordingly, the pulmonary vascular P-Q relationship was shifted to higher pressures at respective flows (Fig. 5a and b). These results demonstrate that ventilation of an isolated perfused mouse lung with hypoxic gas increases PAP.

Baseline perfusion pressure under normoxic ventilation did not differ between LPS-pretreated and untreated mice (control 7.1 ± 0.3 mmHg vs. LPS 7.4 ± 0.3 mmHg, Figs. 4 and 5a). In lungs of control mice, ventilation with hypoxic gas mixture (F_i_O_2_ of 0.01) was associated with an increase of pulmonary pressure (+ 9.2 ± 0.9 mmHg), whereas development of HPV was attenuated in mice with endotoxemia (+ 3.0 ± 0.7 mmHg) after 6 min of hypoxic ventilation (Fig. 4).

Taken together, this data shows that hypoxia induces HPV in lungs of control mice and endotoxemia attenuates HPV.

### Effects of PNU-37883A on pulmonary vascular tone and HPV after lipopolysaccharide challenge

**Fig. 6:**
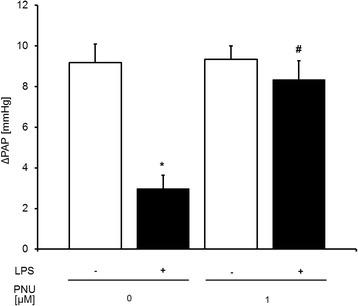
Perfusion with PNU 37883A augments HPV in endotoxemic mouse lungs. Effects of perfusion of lungs obtained from LPS-treated (■) and untreated control (□) mice with 1 μM PNU-37883A upon hypoxia. HPV is expressed as increase in percent of baseline perfusion pressure (∆PAP), (*n* = 8 for control and *n* = 7 for LPS, *P < 0.05 vs. control (w/o LPS and w/o PNU 37883A), †*P* < 0.05 vs. lps. Mean ± SEM, tested with ANOVA, for homogeneity and post hoc Tukey)

To determine whether pharmacological inhibition of Kir6.1 would enhance HPV after exposure to LPS, lungs of mice with and without endotoxemia were perfused with a buffer containing 1.0 μM PNU-37883A. In untreated control mice, inhibition of Kir6.1 by PNU-37883A at a dose of 1.0 μM did not affect baseline PAP under normoxic conditions (control 7.1 ± 0.3 mmHg vs PNU 6.6 ± 0.3 mmHg). Again, rise of PAP started within 2 min and reached its maximum within 6 min. Perfusion with the inhibitor PNU-37883A did not augment HPV in control mice (Fig. 6).

In lungs of endotoxemic mice, inhibition of Kir6.1 by PNU-37883A again did not affect baseline PAP (LPS 7.4 ± 0.3 mmHg vs LPS/PNU 6.6 ± 0.8 mmHg). In contrast, inhibition of Kir6.1 in lungs of endotoxemic mice augmented HPV compared to lungs perfused without inhibitor (Fig. 6).

This shows that PNU-37883A does not alter HPV in control but augments HPV in endotoxemic mice.

### Analysis of pressure-flow curves

A four point pulmonary vascular P-Q curve was recorded in order to obtain more insight in the pulmonary vascular response (Fig. 5a and b). Quantification in the shape of P-Q curves was calculated according to the collapsible vessel model of Permutt and Riley, as discussed and published before [15]. In this ohmic-Starling resistor model, changes in the shape of the P-Q curve are displayed in the slope (R_LIN_) and the extrapolated pressure intercepts at zero flow (P_ZF_) of a linear regression line (Fig. 5c and d) [15–18].

Hypoxia induced an increase of P_zf_ (normoxia 4.0 ± 0.5, hypoxia 8.4 ± 0.6 mmHg) as well as R_LIN_ (normoxia 62.5 ± 3.7 vs hypoxia 128.7 ± 7.5 mmHg*ml-1*kg-1*min-1), thus increasing parallel resistance and critical closing pressure (Fig. 5c and d). Endotoxemia did not alter P_zf_ or R_LIN_ in normoxic condition (P_zf_ normoxia LPS 4.2 ± 0.2 vs control 4.0 ± 0.5 mmHg and R_LIN_ normoxia LPS 61.5 ± 3.7 vs 62.5 ± 3.7 mmHg*ml-1*kg-1*min-1) but resulted in a decrease in P_zf_ and R_LIN_ in hypoxia compared to lungs from healthy animals (P_zf_ hypoxia LPS 5.4 ± 0.4 vs control 8.4 ± 0.6 mmHg and R_LIN_ hypoxia 99.4 ± 9.4 vs control 128.7 ± 7.5 mmHg*ml-1*kg-1*min-1). Perfusion of control lungs with 1 μmol PNU-37883A did not alter P_zf_ in normoxia as well as P_zf_ or R_LIN_ in hypoxia, while decreasing R_LIN_ in normoxia (P_zf_ normoxia 4.4 ± 0.2 vs hypoxia 9.3 ± 0.5 mmHg and R_LIN_ normoxia 49.3 ± 3.1 vs hypoxia 119.5 ± 7.6 mmHg*ml-1*kg-1*min-1). Perfusion of lungs from endotoxemic animals with 1 μmol PNU-37883A resulted in an increase of P_zf_ and R_LIN_ during hypoxia (LPS PNU hypoxia 7.2 ± 1.3 and R_LIN_ hypoxia 139.6 ± 7.8 mmHg*ml-1*kg-1*min-1).

Taken together analysis of pressure flow curves resulted in an increase of P_zf_ (extrapolated pressure intercepting at zero flow) and R_LIN_ (slope of the PQ-curve) in control as well as endotoxemic lungs during hypoxic ventilation. Increase of P_zf_ and R_LIN_ was reduced in lungs of endotoxemic animals and R_LIN_ was increased in PNU-perfused endotoxemic lungs.

Lung wet/dry weight ratios revealed no significant difference between any of the studied groups.

(control 0.11 ± 0.04; LPS 0.11 ± 0.03, PNU 0.10 ± 0.01; LPS/PNU 0.10 ± 0.01, mean ratio ± SEM).

## Discussion

In the present investigation we studied the role of the ATP dependent potassium channel Kir6.1 on HPV in an endotoxemic mouse model. In lungs of endotoxemic mice, Kir6.1 gene as well as protein expressions were increased compared to controls. Immunohistochemical staining showed Kir6.1 expression in small pulmonary vessels. HPV was decreased in endotoxemic animals. Selective inhibition of up-regulated Kir6.1 with PNU 37883A increased HPV in endotoxemic mouse lungs, whereas HPV was unchanged in controls. Our results suggest that LPS induced impaired HPV in mice can be restored by inhibition of overexpressed Kir6.1.

Involvement of the ATP-dependent potassium channel Kir6.1 in systemic circulation has been demonstrated in numerous studies. Kir6.1 expression is up-regulated in rats in LPS as well as in cecal ligation peritonitis (CLP) models [11, 19, 20]. This is not only restricted to rats but has been observed in mouse aortic smooth muscle cells and guinea pigs [6, 21]. Thus, increased Kir6.1 expression is a specific pathophysiologic response to a broad inflammatory stimulus at least in rodents. Endotoxin induced increase of Kir6.1 gene expression in rat mesenteric arteries contributes to systemic hypotension and can be restored by specific inhibition [6, 20].

Besides systemic circulation, Kir6.1 expression is also increased in coronary arteries of endotoxemic mice, leading to vasodilatation and maintaining myocardial perfusion [11, 14]. In Kir6.1 knock out mice, coronary blood flow during endotoxemia is decreased, with reduced cardiac function and augmented mortality [14]. These observations show the pivotal role of Kir6.1 in the regulation of vascular tone, with vasodilatation and hypotension in case of too much Kir6.1 activity and vasospasm in case of total lack in Kir6.1 knock out mice. Thus, inhibition of Kir6.1 increases HPV and by this oxygenation, but complete suppression of Kir6.1 activity is likely to give rise to vasospasm with negative outcome.

Overactivity of Kir6.1 in vascular smooth muscle cells was investigated in transgenic mouse models, showing lower vascular contractility and blood pressure [22]. Besides overexpression of Ki6.1, activation of Kir6.1 in endotoxemia was shown by elevated extracellular arginine levels as well as phosphorylation via inhibited activity of calcineurin [22–25]. Taken together, these studies show an important role of Kir6.1 in vascular contractility and an up-regulation of Kir6.1 expression in endotoxemia in various animal models. This is in line with our data of elevated pulmonary Kir6.1 RNA and protein levels after LPS exposure.

LPS-induced systemic hypotension in rats was attributed to BKCa channel activity as well as KATP activity [6, 26–28]. Although inhibition of KATP channels restored hypotension in numerous animal models of sepsis, administration of the KATP inhibitor glibenclamid in patients with septic shock showed no hemodynamic benefit or increased pulmonary artery pressures but lower blood glucose levels [28, 29]. This might be due to the enteral route of administration of glibenclamid with inadequate plasma concentrations or nonspecific inhibition of SUR rather than specific inhibition of overexpressed pore forming Kir6.1 [6, 29].

Blocking pore-forming subunits of Kir6.1 with PNU-37883A showed an inhibitory potency on native KATP currents in pig urethral myocytes as well as in rat mesenteric arteries [29]. The concentration of PNU-37883A used in our experiment is in line with an effective dose of 1.1 μM PNU-37883A in isolated rat mesenteric arteries [9] and pig urethral smooth muscle cells (0.7 μM) [29]. Tomoda found a maximal vasoconstrictive effect of 3 μM PNU-37883A in levcromakalim (a Kir6.1 channel opener) relaxed pig urethral smooth muscle cells but the opposite effect with higher doses of PNU-37883A in Bay K 8644 (a L-type calcium channel activator) treated smooth muscle cells [29]. This was explained by voltage dependent inhibition of KATP currents by PNU-37883A [29]. Selective inhibition of Kir6.1 with 1 μM PNU-37883A of denuded aortic rings 18 h after CLP decreased resting membrane potential in CLP but not in control rings and increased basal tension in CLP but not in control rings [20]. This shows a higher sensibility to PNU-37883A suggesting an up-regulation of Kir6.1. Since this was observed in denuded aortic rings, it indicates that the effect is at least in part independent from the endothelium. This might be relevant since Kir6.1 is also expressed in some endothelial cells.

Besides KATP channels, inhibitory effects of PNU-37883A on voltage-dependent Ca^2+^ channels were observed in a rat model of pinacidil (a cyanoguanidine compound opening ATP-modulated potassium channels) induced hypotension with selectivity for Kir6.1 and less affinity to pancreatic Kir6.2 [30–32]. The fact that PNU-37883A did not augment further the magnitude of HPV in control mice may be due to less activity of Kir6.1 in controls compared to LPS treated animals or an already maximal vasoconstrictor response by ventilating lungs with 1% oxygen.

Immunohistochemistry showed Kir6.1 immunoreactive protein in small pulmonary vessels. Besides arteries, veins can also contribute to total pulmonary vascular resistance [33–35], an effect that is dependent on age and species. In agreement to our data, Michelakis reported expression of Kir6.1 gene expression in pulmonary arteries and veins of rats [35]. Taken together, expression of Kir6.1 is not restricted to systemic circulation but also can be found in lung vessels.

LPS increased Kir6.1 expression may be mediated by NFκB. It has been reported that NFκB regulates Kir6.1 expression and in turn expression of NFκB is dependent on the nitric oxide pathway [19]. Involvement of the nitric oxide pathway in endotoxemia is also supported by studies restoring HPV through inhibition of inducible nitric oxide synthetase (iNOS) [15] or soluble guanylate cyclase (sGC) [36]. Induction of Kir6.1 expression via sGC has also been reported in a guinea pig ileitis model, when mesenteric lymphatic pumping was compromised while iNOS and Kir6.1 gene expression were up-regulated [21]. Inhibition of iNOS, sGC or Kir6.1 in isolated, luminally perfused mesenteric lymphatic vessels improved lymphatic pumping [21]. NFκB expression can by down-regulated by CO in LPS stimulated mouse peritoneal macrophages [37]. Thus, restoration of HPV by inhaled CO (iCO) in endotoxemic mice [38] might be attributable to anti-inflammatory effects of iCO via NFκB expression.

Taken together, induced Kir6.1 expression and activity by increased NFκB expression (via nitric oxide and sGC) is a possible pathway to reduce HPV in endotoxemia.

On cellular level, activated Kir6.1 might decrease HPV via increased efflux of K^+^ and thereby decreased intracellular Ca^2+^. Selective inhibition of overexpressed Kir6.1 results in a decrease of K^+^ efflux which in turn increases Ca^2+^-influx and smooth muscle cell contraction, resulting in increased HPV. Voltage gated potassium channels involved in HPV of non-endotoxemic animals are Kv1.5 and Kv2.1 [39]. A number of tested voltage gated potassium channels (Kv1.5, Kv2.1, Kv3.1) were unchanged on RNA level in lungs of endotoxemic mice [3]. On the contrary, Kv1.2 was found to be less expressed in endotoxemia [3]. Analysis of pressure-flow relationships are in line with previous studies, with hypoxia-induced increases in mean parallel ohmic resistance R_LIN_ as well as an increase of extrapolated closing pressure P_ZF_ in controls [15]. These hypoxia-induced increases in R_LIN_ and P_ZF_ were reduced in endotoxemia compared to untreated mice as described before [15]. Perfusion with PNU-37883A did not change normoxic P_ZF_ and showed same patterns as controls during hypoxic ventilation. In contrast, perfusion of endotoxemic mouse lungs with PNU-37883A increased both, R_LIN_ and P_ZF_, thus antagonizing LPS induced decreased pulmonary vascular resistance as well as selectively enhancing vasoconstrictor response upon hypoxia. In a similar LPS mouse model with in vivo measurements, reduced HPV was observed in endotoxemia, resulting in a V/Q mismatch and systemic hypoxia [35]. Due to the model of the isolated perfused mouse lung, we were not able to measure the parameter of systemic oxygenation. Instead, we measured the total increase of pulmonary pressure upon hypoxia.

Up-regulation of Kir6.1 gene expression in endotoxemic mice is consistent with data from mouse smooth muscle cells showing a transcription dependent up-regulation after 20 h of LPS exposure [11]. Like Weiwei, a LPS model of endotoxemia was used since it shows a high reproducibility of systemic inflammation with less variability compared to cecal ligation and puncture models [39]. The model of the isolated perfused mouse lung offers a possibility to study selective effects of pulmonary vasoactive drugs and hypoxic stimuli without interfering with other organs like the heart, which might be affected by the studied drug by arrhythmias or a modified cardiac output [3]. Since the model excludes systemic circulation, parameter like systemic oxygenation cannot be measured. In vivo measurements in mice showed a decrease of systemic oxygenation and a V/Q –mismatch in a similar LPS i.p. model also used in this study [40].

In order to exclude acute cyclooxygenase dependent mechanisms to interfere with HPV, the cyclooxygenase inhibitor indomethacin was added to the perfusate. Metabolism of arachidonic acids by cyclooxygenases in the lung produces diverse prostaglandins, regulating vascular tone. Loss of HPV as well as systemic vasodilatory effects can be attributed to these prostaglandins. The cyclooxygenase inhibitor indomethacin was shown to increase HPV in a canine model of lung injury and to reduce HPV in isolated pulmonary sheep veins but had no effect in a murine model of HPV [41–43].

Nitric oxide produced by NOS (nitric oxide synthetase) regulates pulmonary vascular tone. Since we wanted to exclude acute NOS dependent effects in our model, perfusion buffer contained L-NAME. Inhibition of NOS by L-NAME augments HPV dose-dependent in blood perfused isolated rat lungs, whereas it has no effect on buffer perfused rat lungs [44, 45]. There was no change by L-NAME in baseline perfusion pressure in buffer perfused isolated perfused rabbit or mouse lungs [46, 47].

Lung wet/dry weight ratios were comparable between groups, suggesting the absence of pulmonary edema. This is in contrast to in vivo observations of endotoxemic mice, where lung edema was found to be iNOS-dependent [48]. However, our data is in line with previous studies of endotoxemic isolated perfused mouse lungs, that used the same perfusate containing albumine 5% as well as dextran 5% [3, 15, 16]. Thus, the in vitro model is able to compare effects of hypoxia or pharmacological inhibition from the same starting PAP in control and endotoxemic lungs but might display other results than in vivo measurements of acute lung injury. Another factor might be the type of LPS used, since induction of iNOS expression can vary even with the LOT number of [16].

## Conclusion

The ATP sensitive K-channel Kir6.1 is overexpressed in lungs of LPS treated mice on RNA as well as protein level and can be inhibited by PNU-37883A, thereby augmenting HPV in endotoxemia but not in controls. Our study suggests that selective inhibition of Kir6.1 might represent a useful tool to enhance HPV in subjects with endotoxemia and respiratory failure.

## Abbreviations

ARDS: Acute respiratory distress syndrome
bw: Body weight
EGTA: Ethylene glycol-bis (2-aminoethylether)-N,N,N′,N′-tetraacetic acid
F_i_O_2_: Fraction of inspiratory oxygen
GAPDH: Glyceraldehyde 3-phosphate dehydrogenase
HPV: Hypoxic pulmonary vasoconstriction
i.p.: Intra peritoneal
iCO: Inhaled carbon monoxide
iNOS: Inducible nitric oxide synthetase
KATP: ATP-regulated potassium channels
Kv: Voltage-gated potassium channel
L-NAME: Nω-Nitro-L-arginine methyl ester hydrochloride
LPS: Lipopolysaccharide
mRNA: Messenger ribonucleic acid
NFκB: Nuclear factor ‘kappa-light-chain-enhancer’
NOS: Nitric oxide synthetase
PAP: Pulmonary arterial pressure
PBS: Phosphate-buffered saline
P_ZF_: Mean critical closing pressure
RBC: Red blood cell
R_LIN_: Mean parallel resistance
rRNA: Ribosomal ribonucleic acid
sGC: Soluble guanylate cyclase
SUR: Sulfonylurea receptor subunit
